# Jorvik: A membrane-containing phage that will likely found a new family within Vinavirales

**DOI:** 10.1016/j.isci.2023.108104

**Published:** 2023-09-29

**Authors:** Pavol Bárdy, Conor I.W. MacDonald, Roman Pantůček, Alfred A. Antson, Paul C.M. Fogg

**Affiliations:** 1 York Structural Biology Laboratory, Department of Chemistry, University of York, Heslington, York YO10 5DD, UK; 2 Department of Experimental Biology, Faculty of Science, Masaryk University, 625 00 Brno, Czech Republic; 3 York Biomedical Research Institute, University of York, Wentworth Way, York YO10 5NG, UK; 4 Biology Department, University of York, Wentworth Way, York YO10 5DD, UK

**Keywords:** Virology, Ecology, Microbiology

## Abstract

Although membrane-containing dsDNA bacterial viruses are some of the most prevalent predators in aquatic environments, we know little about how they function due to their intractability in the laboratory. Here, we have identified and thoroughly characterized a new type of membrane-containing bacteriophage, Jorvik, that infects the freshwater mixotrophic model bacterium *Rhodobacter capsulatus*. Jorvik is extremely virulent, can persist in the host integrated into the RuBisCo operon and encodes two experimentally verified cell wall hydrolases. Jorvik-like prophages are abundant in the genomes of Alphaproteobacteria, are distantly related to known viruses of the class *Tectiliviricetes,* and we propose they should be classified as a new family. Crucially, we demonstrate how widely used phage manipulation methods should be adjusted to prevent loss of virus infectivity. Our thorough characterization of environmental phage Jorvik provides important experimental insights about phage diversity and interactions in microbial communities that are often unexplored in common metagenomic analyses.

## Highlights

• Jorvik is a circular dsDNA membrane-containing phage distinct from model phage PM2
• Jorvik-like prophages are abundant in genomes of Alpha-proteobacteria
• Host regulator CtrA and recombinase XerCD are likely required for the life cycle
• Spontaneous lytic variant clears host cultures at multiplicity of infection <0.01

## Graphical abstract

**Figure:**
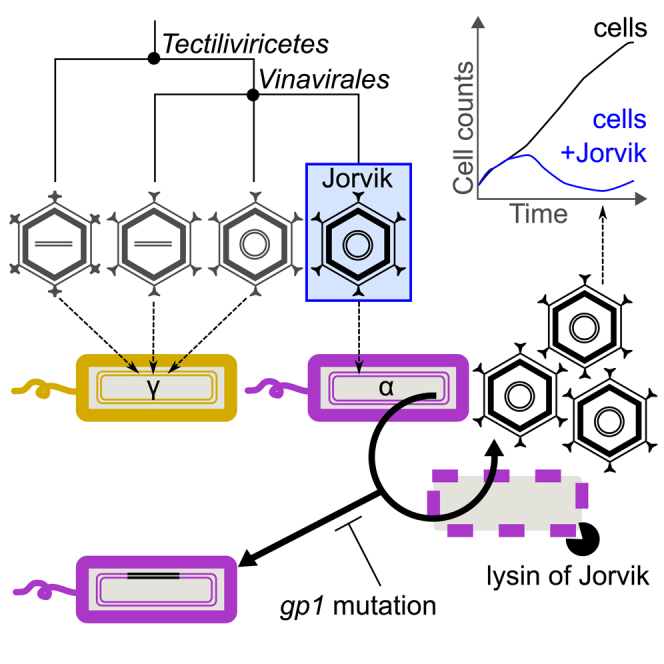

## Introduction

Non-tailed bacteriophages are an understudied group of bacterial viruses;^1^^,^^2^^,^^3^ an issue rooted in problems with sensitivity to chloroform, presence of proteins covalently bound to genomic DNA that hinder genome isolation and purification bias toward identification of tailed phages, e.g., non-tailed phages have a different buoyant density than used by most established protocols.^4^^,^^5^^,^^6^^,^^7^ Nevertheless, recent microscopic, metagenomic and bioinformatic studies have revealed that non-tailed viruses are abundant in the environment, which emphasizes their potential importance and thus the necessity for further research.^5^^,^^8^^,^^9^

Membrane-containing double-stranded (ds)DNA viruses with double jelly-roll fold capsids belonging to the PRD1-adenoviral lineage, *Bamfordvirae* kingdom, infect all domains of life.^10^^,^^11^^,^^12^ Bacteria-infecting viruses of this kingdom belong to the class *Tectiliviricetes* and include some of the most prevalent predators of aquatic environments.^5^^,^^8^ The protein shell of *Tectiliviricetes* phages is reinforced by its anchoring into an internal membrane, which is likely to provide extra stability against mechanical stress.^13^ Based on the genome type, gene synteny and capsid characteristics, phages of this class are split into three families: *Tectiviridae, Autolykiviridae,* and *Corticoviridae*.^14^

Out of the three families, *Tectiviridae* is the best-studied. Phages belonging to the *Tectiviridae* contain a linear dsDNA genome and create a transient membrane protrusion with a characteristic tubular shape for genome delivery.^15^ Linear dsDNA phages belonging to the *Autolykiviridae* and *Tectiviridae* differ in their respective virion proteins, with the former being structurally similar to those of *Corticoviridae* phages. Unlike other phages of the lineage, *Autolykiviridae* phages often possess a broad host range, dominating the viral infection network of *Vibrionaceae* species.^5^ In contrast to these two families, phages belonging to the *Corticoviridae* contain a circular dsDNA genome. Members of this family have been shown to infect marine bacteria^16^ but only one, pseudoalteromonal phage PM2, has been studied in detail.^1^^,^^4^^,^^7^^,^^17^^,^^18^ Interestingly, double jelly-roll fold capsids may combine even with ssDNA replication modules,^19^^,^^20^ and much of the lineage diversity remains to be explored.

In this study, we identified a novel membrane-containing circular dsDNA phage, Jorvik – named after the Viking designation of the city of York where this phage was described. Jorvik is highly virulent and homologous prophages are abundant in proteobacterial genomes. We performed experimental validation for several phage components, critical for understanding how this group of phages function. Phylogenetic analysis suggests that Jorvik-like phages form a new family-level taxon within the *Tectiliviricetes* that shares a common ancestor with the *Autolykiviridae* and *Corticoviridae* families as well as recently described fNo16-like phages.^21^ We propose that all these phages should be classified in the same order, *Vinavirales*, and we refer to them as such in the rest of the manuscript. When working with an active virus stock, our experimental process focused on the constant propagation of Jorvik with minimal culture manipulation. We suggest that this approach can be applied to the cultivation and characterization of other membrane-containing dsDNA viruses, allowing their hitherto unseen diversity to be revealed.

## Results

### Origin of phage jorvik and its ability to spontaneously form more virulent variants

When cultivated in a defined RCV liquid medium, we observed that the *Rhodobacter capsulatus* B10 strain spontaneously produced bacteriophage particles capable of infecting the *R. capsulatus* SB1003, YW1, DE442 and St. Louis strains when they were grown in a rich YPS medium (Table 1). However, even for a relatively short time, the phage particles were unstable in liquid media, as demonstrated by a 90% decline in infective titer after just 24 h of incubation in YPS (Data S1.1). The phage was thus routinely propagated on YPS agar plates with a soft agar overlay, under aerobic conditions. For long-term storage, pieces of soft agar were taken from confluently lysed plates by a sterile inoculation loop, transferred to an Eppendorf tube, frozen and kept at −80°C.

**Table 1:** Interaction between phage Jorvik and the tested host strain

*Rhodobacter capsulatus* strain	Plaque-forming ability^a^	Integrated phage^b^	CRISPR spacers match	*attB* in the bacterial genome	GenBank accession	Reference
SB1003	+	–	–	+	NC_014034	Strnad et al.^22^
DE442	+	–	–	+	NZ_AYPR00000000	Ding et al.^23^
B10	–	+	–	+	JAOTPJ000000000	Weaver et al.,^24^ this study
St. Louis	+	–	–	+	NZ_VIBE00000000	Weaver et al.^24^
St. Louis C1	–	+	–	+	NA	this study
YW1	+	–	–	+	NZ_AYPY00000000	Ding et al.^23^
B6	–	–	+	+	NZ_AYQA0000000	Ding et al.^23^
YW2	–	–	–	+	NZ_AYPZ00000000	Ding et al.^23^
*Rhodobacter sphaeroides* strain 2.4.1	–	–	NA	NA	NA	Leidenfrost et al.^25^
H9	–	–	NA	NA	NA	Weaver et al.^24^
P12F1	–	–	NA	NA	NA	Weaver et al.^24^
SP18	–	–	NA	NA	NA	Weaver et al.^24^
SP36	–	–	NA	NA	NA	Weaver et al.^24^
*Rhodobacter sphaeroides* strain SCJ	–	–	NA	NA	NA	Weaver et al.^24^

See also Table S3.

a same results were observed for variants Jorvik1 and Jorvik2.

b tested by positive PCR amplification of genes Slt and M15, spontaneous induction of YW1-infecting phage particles when cultivated in RCV; +, positive result; -, negative result; NA, data not available.

**Figure 1:**
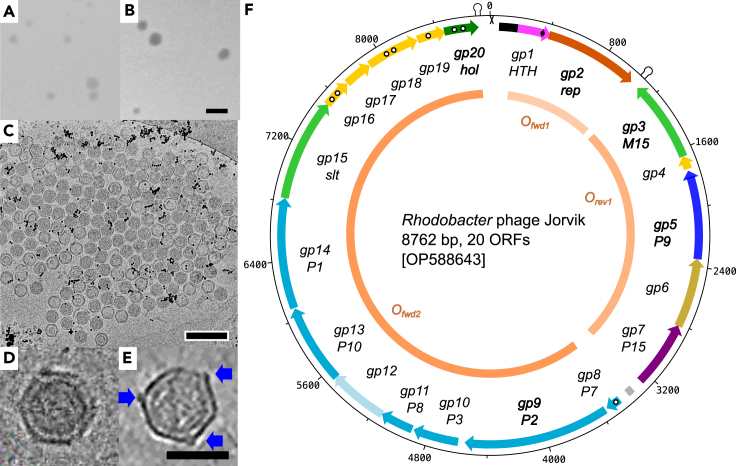
Morphological and genomic characteristics of phage Jorvik (A and B) Morphology of Jorvik1 (A) and Jorvik2 (B) plaques on the lawn of *R. capsulatus*, strain YW1. The scale bar is 2 mm. (C) An aggregate of the phage obtained from a lysed plate wash, imaged by cryo-EM. The black dots are gold fiducials, scale bar represents 200 nm. (D and E) Native capsids of the phage imaged using cryo-EM (D) and cryo-ET reconstruction (E) with head spikes depicted by blue arrows, scale bar is 50 nm. (F) Genome map of the phage variant Jorvik1. The duplicated region of Jorvik2 adjacent to *gp1* is highlighted in black and the single nucleotide change in *gp1* is depicted by black dot. Genes encoding structural proteins (medium blue), acetyltransferase (light blue), hydrolases (light green), unknown function (yellow), holin (dark green), DNA-binding protein (magenta), replicase (orange), ATPase (dark blue) and P15-like repressor (dark magenta) are depicted. Genes shown in bold have sequence homologues in the database, white dots within ORF arrows depict predicted transmembrane regions. Putative regulatory repeat (gray), terminators (loop symbol) and *attP* site (X symbol) are highlighted. Operons are depicted in shades of orange in the center of the map. See also Figures S1‒S3 and Data S2, S4, and S7.

The phage did not form plaques on cells growing under anaerobic conditions nor on aerobic RCV plates. The optimal temperature range for plaque formation on YPS agar plates was 20°C–34°C (Figure S1A). The plaques were 0.1–1.2 mm in diameter and had a turbid halo with a less turbid center (Figure 1A). After several rounds of passage with *R. capsulatus* YW1, a plaque with a turbid halo and a completely clear center appeared (Figure 1B). We designated the phage Jorvik1, for the original turbid plaque variant, and Jorvik2, for the clear plaque variant. It is notable that spontaneous induction of two phages from *R. capsulatus* B10 that were capable of infecting St. Louis was previously observed by Wall et al*.*^26^ These phages were not characterized in any detail; therefore, we were not able to make any comparison to phage Jorvik.

The titer of the phage was completely lost when the lysate was treated with chloroform, which is indicative of membrane-containing phages. Surprisingly, the titer declined substantially during repeated rounds of centrifugation and was negatively affected by filtration (Data S1.2). Cryo-EM imaging showed that phage particles extracted from confluently lysed plates were present in tightly packed formations rather than as individual virions (Figure 1C), with most of the grid being free of particles. Tight arrangements have been observed before when viral particles are at high density,^27^ however, here Jorvik only reached relatively low titers in the range of 10^6^ to 10^7^ pfu mL^−1^. These results suggest that in YPS media, Jorvik virions may behave as larger aggregates.

### The Jorvik genome and virion morphology are characteristic of the *Tectiliviricetes*

The Jorvik virion is an icosahedron with approximately 59 nm diameter, decorated with short spikes and containing a spherical density most likely corresponding to an inner membrane (Figures 1D and 1E). The genome of Jorvik1 consists of 8,762 bp circular dsDNA (Figure 1F). The GC content is 62.4%, which is similar to the 66.6% GC of the host strain *R. capsulatus* SB1003.^22^ We identified 20 ORFs in the phage genome and predicted their function (Tables S1 and S2, Figure S2, and Data S2). The ORFs are encoded in three distinct operons, two on the forward strand and one on the reverse strand.

The 8,889 bp genome of Jorvik2 differs from that of Jorvik1 in two loci. The first is a duplication of a non-coding region (position 60–186) located between the phage integration attachment site, *attP,* and a gene predicted to encode a helix-turn-helix (HTH) domain-containing protein, Gp1. The second difference is a single nucleotide change inside the gene encoding Gp1 that results in a W56L single amino acid substitution. Since both differences were mapped within or near *gp1*, we conclude that the product of this gene plays a role in the lysis-lysogeny decision.

We did not detect tubulation morphologies resembling those reported for *Tectiliviridae* and *Autolykiviridae* phages, either for Jorvik particles in solution or attached to cell membranes.^5^^,^^15^ Instead, we observed particles with ruptured virions from which DNA was escaping and cell membrane-attached particles that seem to lack part of the capsid at the attachment site (Figures S1B‒S1G). It would be speculative to conclude that this rupturing is of biological significance, but nevertheless, these observations agree with the previously proposed hypothesis that the genome delivery mechanism of membrane-containing circular dsDNA phages differs from that of membrane-containing linear dsDNA phages.^28^

### Phage Jorvik1 integrates into the RuBisCO operon

The turbid zone of phage plaques is often caused by the integration of phage DNA into the genome of the host bacterial cells, resulting in lysogenic immunity against superinfection.^29^^,^^30^ To test if this was also the case for Jorvik1, the phage was plated on *R. capsulatus* St. Louis, which produced the most turbid plaques. The turbid zone of a single plaque was picked with a sterile tip and passaged on fresh plates to propagate phage-resistant mutants. Three individual colonies were subsequently tested for spontaneous induction of the phage, for resistance to Jorvik infection, and for the presence of Jorvik genes *gp3* (*M15)* and *gp15* (*slt)* by PCR (Data S3.1). One of the three colonies tested, designated St. Louis C1 hereafter, produced positive results for all three tests. After three passages, 10/10 St. Louis C1 colonies remained positive for the presence of the virus, suggesting stable maintenance in the host. Interestingly, we were unsuccessful in isolating a stable lysogen of the Jorvik2 variant when employing the same approach, testing 20 colonies from different susceptible *R. capsulatus* strains.

Genomic DNA of both the parental *R. capsulatus* B10 strain and the lysogenic St. Louis C1 strain was isolated and sequenced to identify the integration site of the phage. In both St. Louis C1 and B10, the phage was integrated into the bacterial genome within the RuBisCO *cbbII* operon between the genes encoding NAD(P)H-dependent quinone oxidoreductase (*rcc01836*) and phosphoglucomutase (*rcc01837*). Jorvik does not possess a predicted integrase gene but the *attP* and *attB* sequences show similarity to *dif* motifs recognized by a cellular XerCD recombinase system,^31^^,^^32^ with the motif being conserved among Jorvik-like phages (Figure S3A). The actual location of the *attB* in the genomes of bacteria containing Jorvik-like prophages seems variable. It was recently hypothesized that the host XerCD is utilized for the integration of gammaproteobacterial membrane-containing dsDNA phages.^21^ Our results suggest the same mechanism for Jorvik-like phages.

### Phage Jorvik has a narrow host range and requires the pleiotropic regulator CtrA for infection

Phages Jorvik1 and Jorvik2 had identical host ranges for the *R. capsulatus* strains tested here (Table 1). Jorvik infected closely related strains, all of which had intact *attB* sites in their genome (Table 1). Apart from the original B10 source strain and the St. Louis C1 lysogen, PCR amplification of the *M15* and *slt* genes did not produce a product for any other strain tested (Data S3.1); this suggests that there are no other lysogens of Jorvik among these strains. Bioinformatic analysis revealed the presence of CRISPR-Cas spacers matching the phage in diverse *R. capsulatus* isolates. These are strain A12 isolated in China, A52 and B41 isolated in Turkey and B6 isolated in the USA. Here, the B6 strain was shown to be resistant to infection by Jorvik (Table 1), suggesting that the system is functional.

To identify host genes that are required for successful phage infection, six available *R. capsulatus* SB1003 gene knockout strains were tested against both Jorvik1 and Jorvik2 (Table S3).^33^ Of the six strains, only one carrying a deletion of the pleiotropic regulator *ctrA* was consistently resistant to infection. The involvement of CtrA was verified by successful restoration of infection by *in trans* complementation with a *ctrA* plasmid (Table S3). The correct phosphorylation state of CtrA seems to be important for the phage infection because the phospho-null (D51A) and phospho-mimetic (D51E) forms do not fully complement the wildtype product; a similar effect was observed for *R. capsulatus* gene transfer agent production.^34^ We identified one putative CtrA-binding site, located in the intergenic region between the structural and packaging operon near a putative regulatory repeat (Figure S3B). It has been demonstrated that CtrA binds to the regulatory regions of several temperate-tailed alphaproteobacterial phages.^35^ Our results support the hypothesis that CtrA is widely involved in the regulation of lysogenic phages in the Alphaproteobacteria.

### Growth characteristics show that phage Jorvik2 is highly virulent

**Figure 2:**
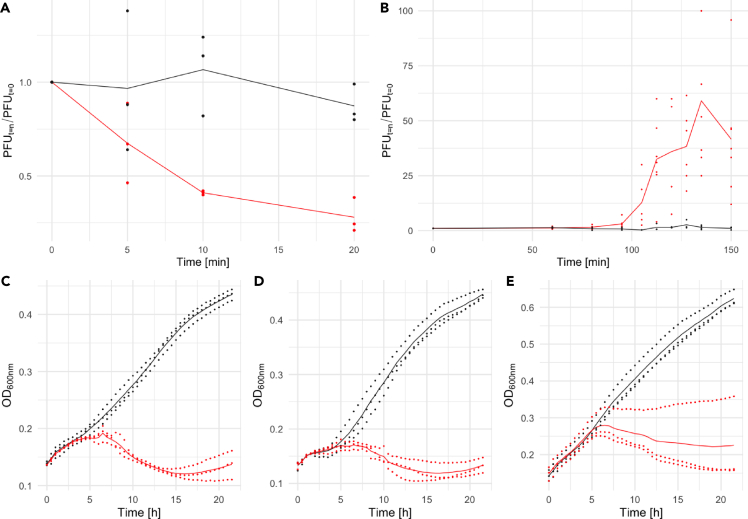
Growth characteristics of phage Jorvik2 (A) Adsorption curve on the propagating strain YW1 (red) and phage-resistant strain P12F (black). On the y axis, values of plaque-forming units (PFUs) for specific time points relative to PFUs at time point 0 are shown. (B) Growth curve of the phage on the propagating strain YW1 (red) and sterile media control (black). On the y axis, PFUs for specific time points relative to PFUs at time point 0 are shown. (C‒E) Growth curves of strains YW1 (C), SB1003 (D) and St. Louis (E) with (red) and without the phage (black) are shown. The multiplicity of infection was below 0.01. The raw data are present in Data S1.3–S1.5.

Phage Jorvik adsorption kinetics followed a linear trend until approximately 60% of particles had adsorbed; the adsorption rate for this 10 min post-infection period was equal to 4.9 × 10^−10^ mL min^−1^ (Data S1.3). This adsorption rate suggests that the phage recognizes 10–100 receptors per host cell.^36^ Over a more prolonged adsorption period, a subfraction of slower-adsorbing phage was identified. The adsorption efficiency of Jorvik reached 72% by the 20 min post-infection time point (Figure 2A). Inefficient adsorption of a sub-population is common in phages^37^ and was reflected in the relatively broad rise period observed in the phage growth curve (Figure 2B). The latent period of the phage is 80–95 min and the titer peaks at ∼130 min post-infection, with the inflexion point of the curve at around 110 min. These times are longer in comparison to *Alphatectivirus* phages infecting faster-growing *E. coli* (inflexion point ∼45 min)^38^ or the *Corticovirus* phage PM2 that infects *Pseudoalteromonas* (∼70 min),^4^ but shorter than that of tailed Roseobacter phage RDJL phi1 (∼140 min).^39^ To our knowledge, apart from Jorvik, RDJLphi1 is the only *Rhodobacteraceae*-infecting phage with an estimated growth curve and more microbiological data are required to assess what variability in propagation speed exists among these phages. For the growth conditions tested, the burst size of Jorvik was estimated as 45 ± 27 phage particles per infected cell (Data S1.4).

Jorvik phage can effectively suppress growth of all three *R. capsulatus* propagation strains – YW1, SB1003 and St. Louis (DE442 was not tested as it is an SB1003 derivative). With an initial multiplicity of infection (MOI) in the range of 0.0005–0.005 (Data S1.5), the bacterial density starts to decline 5–6 h post-infection (Figures 2C–2E). Based on the phage growth curve, we estimate this as the end of the third lytic cycle of the phage. After >20 h of incubation, phage-resistant cell growth is noticeable in all three strains (Figures 2C–2E). Since the estimated MOI required for the clearance of the cultures was strikingly low, we tested the effect of cell growth state and the choice of titer method on the efficiency of phage plating (Data S1.2). The data showed that both variables affected the titer estimation by around 2-fold, adjusting our MOI estimate to 0.001–0.01.

### Phage virions survive repeated freeze-thaw and pH shifts

**Figure 3:**
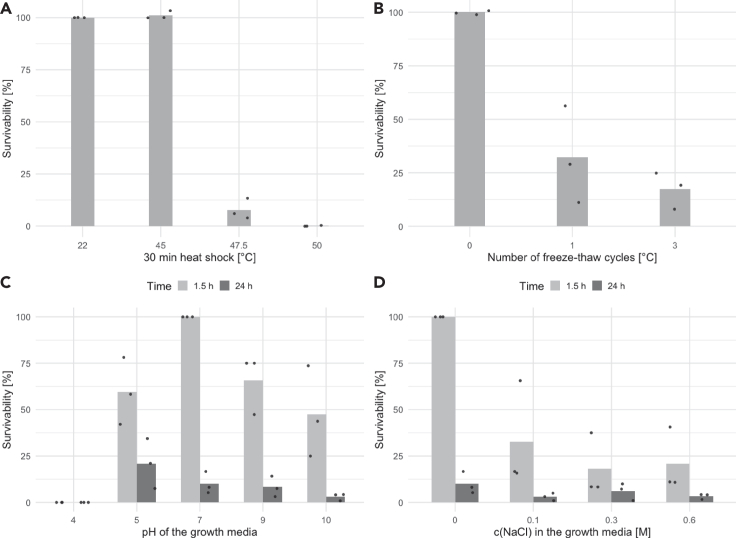
Phage survivability under different conditions Individual measurements of biological replicates are plotted as points, their averages are plotted as Bars. (A) Heating of the phage lysate for 30 min. (B) Freeze-thawing of the phage lysate. (C) Incubation of the phage in the medium with different pH. (D) Incubation of the phage in the medium with different NaCl concentrations. The raw data are present in Data S1.1 and S1.6–S1.8.

The stability of the Jorvik virions in response to temperature, freezing-thawing, pH and salinity was tested. The phage was resistant to 30 min incubation at temperatures up to 45°C, but the titer decreased rapidly when incubated at 47.5°C and 50°C (Figure 3A). In the freeze-thaw experiment the titer dropped to an average of 32% after a single cycle, compared to a control incubated at room temperature (Figure 3B). Additional freeze-thaw cycles had only a minor additional influence on the phage titer. Incubation for 24 h in YPS media at pH 5 to 9 did not affect phage stability compared to the pH 7 control. There was a slightly larger decline in infectivity during incubation at pH = 10 and a complete loss of titer in pH = 4 media, even after 90 min of incubation (Figure 3C). When diluted in media of increasing salinity, the phage titer dropped to 33, 18, and 21% on average for YPS media with 0.1 M, 0.3 M, and 0.6 M NaCl, respectively, compared to YPS media with 0 M NaCl. After the initial osmotic shock, prolonged incubation in high salinity media did not lead to a larger decline of the titer compared to the decline in salt-free media (Figure 3D).

### Proteomic analysis confirmed an abundance of predicted structural proteins in the virion-enriched sample

To assess which gene products are part of the Jorvik virion and indirectly confirm their function, an LC-MS/MS analysis was performed on a partially purified sample of Jorvik2 and the relative abundance of the proteins was estimated (Data S4). The partially purified sample was prepared by differential centrifugation as described in the STAR Methods section. A thorough purification was not possible due to the problems associated with different purification procedures and prolonged storage described above. The structural operon of phage Jorvik shares equivalent synteny and structural homology with the model phage PM2, thus we apply the established phage PM2 nomenclature for the Jorvik gene products.

Gp9, which corresponds to the putative major capsid protein P2, was the most abundant viral protein followed by the putative receptor-binding spike P1 (Gp14) and the putative structural membrane protein P3 (Gp10). Structural proteins P1, P2 and P3 are also the three most abundant proteins in phage PM2 virions.^7^^,^^18^ Another 13 phage gene products were identified in the sample, and all were present in comparable amounts to background host bacterial proteins (Data S4). All 13 products were encoded in the structural and packaging module of the genome. Four putative phage proteins were not detected by the MS analysis. These include the HTH-domain protein Gp1, replicase Gp2, putative holin Gp20 and, unexpectedly, a homologue of phage PM2 structural membrane protein P8 (Gp11). The presence of the very short Gp4 in the sample could not be estimated, as the theoretically acquired peptides after trypsin cleavage would be too short to analyze.

### The phage encodes two lytic enzymes

**Figure 4:**
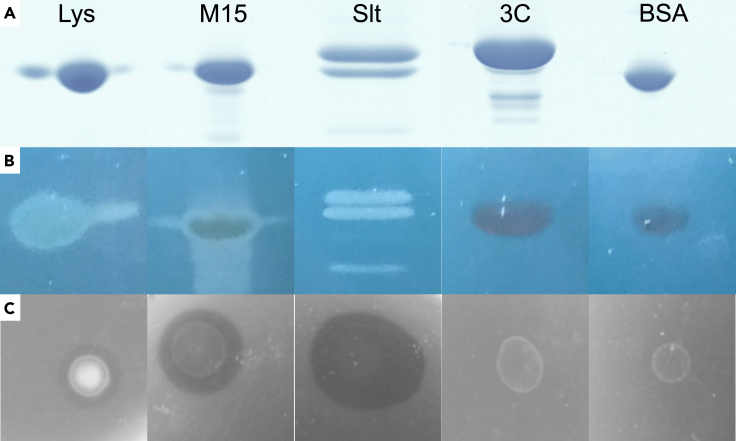
Peptidoglycan hydrolyzing activity of phage-encoded enzymes (A) SDS-PAGE gel of the lytic protein samples – lysozyme positive control (Lys), putative peptidase M15 (M15), putative soluble lytic transglycosylase (Slt) and negative controls protease 3C (3C) and bovine serum albumin (BSA). (B) Zymogram with *R. capsulatus* SB1003 cell walls embedded in the gel matrix. The raw gels are present in Data S3.2. (C) Plate lysis assay of protein samples dropped onto a layer of water agar with embedded cell walls.

Two gene products have predicted similarity to peptidoglycan hydrolases. One designated as Slt is 231 amino acids (AA)-long, located in the structural module, and has a C-terminal domain similar to the lytic transglycosylase P7 from phage PRD1 (HHpred; P27380, E-value = 9.7e-8, Score = 71.57; region 102–225). The second designated as M15 is 184 AA-long, located on the opposite strand packaging locus and is similar to peptidases from the M15 family (HHpred; PF08291.14, E-value = 1.4e-21, Score = 137; region 24–129). To confirm their function in *R. capsulatus* cell wall digestion, the genes encoding these products were cloned, overexpressed and the proteins purified using his-tag affinity chromatography. Both purified proteins showed *R. capsulatus* cell wall degradation activity in zymogram and plate lysis assays (Figure 4, Data S3.2).

### Phylogenetic analysis classifies Jorvik-like phages as a novel family-level group within the *Vinavirales* order

**Figure 5:**
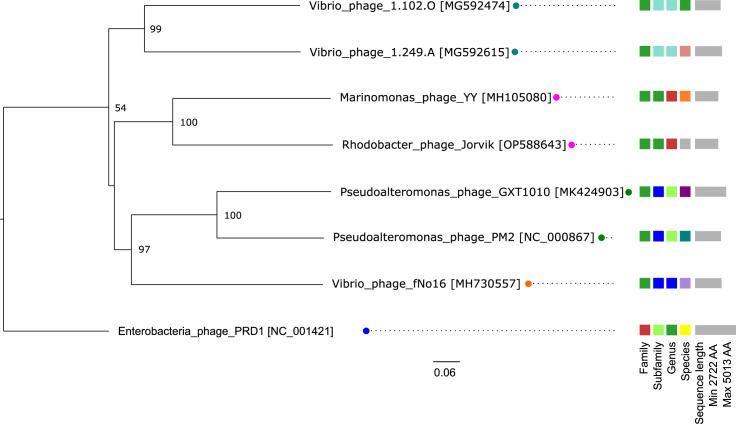
Phylogenetic tree of phage Jorvik generated by Genome-BLAST Distance Phylogeny of the predicted amino acid sequence using the web server VICTOR The pseudo-bootstrap support values from 100 replications are shown next to nodes, with an average support equal to 90%. The scale bar defines the branch length, scaled in terms of the D6 distance formula. The legend on the right defines different taxonomy rankings of the phages with the color coding differentiating between different taxon groups that were assigned within a rank. The color of the name depicts approved or officially proposed family ranks – *Autolykiviridae* (teal), Jorvik-like (magenta), *Corticoviridae* (green), fNo16-like (orange) and *Tectiviridae* (blue). See also Table S4 and Data S5 and S6.

**Figure 6:**
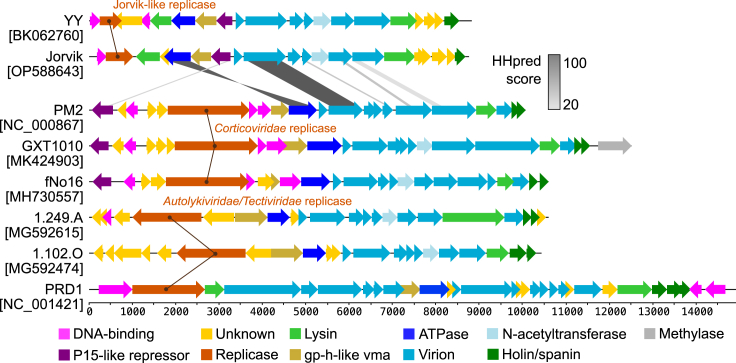
Genome synteny of membrane-containing dsDNA bacteriophages Names of the phages with GenBank accession numbers are shown on the left. The color bar on the right shows HHpred score for the pairwise alignment between corresponding homologues of Jorvik and PM2 phage, with raw values present in Table S2. The scale bar at the bottom shows the length of the genome in base pairs. Phages are grouped according to the replicase gene type. Genes are colored according to their putative function as explained in the legend.

Phylogenetic analysis of two conserved proteins, the packaging ATPase P9 (Gp5) and the major capsid protein P2 (Gp9) (Data S5), as well as Genome-BLAST Distance Phylogeny,^40^ showed that phages Jorvik and *Marinomonas* phage YY form a unique family-level group within the *Vinavirales* (Figure 5). This is consistent with the level of differences observed during the manual inspection of genome synteny among the phages of *Vinavirales* (Figure 6). The major distinction between Jorvik and other phages of *Vinavirales* lies in a different set of regulatory genes, the replicase gene and lytic genes. The synteny of structural and packaging loci is more conserved, with several Jorvik proteins identified as homologous phage PM2 counterparts using HHpred^41^ (Table S2).

To estimate the prevalence of prophages similar to Jorvik integrated into sequenced bacteria genomes, PSI-BLAST bioinformatic analysis of the two most conserved proteins, P9 and P2, was used to identify sequences with both products encoded within a 10 kb window. In total, 849 prophage hits were identified, belonging to 737 unique bacterial strains and 436 unique bacterial species (Data S6.1). These prophages were found predominantly in aquatic Alpha, Beta and Gammaproteobacteria species (Table S4). We manually inspected contig sequences from twelve selected representatives spread throughout the taxonomic lineages, and confirmed that they all encoded additional PM2-like proteins in the P9/P2 locus (Table S4). Clustering of P9 and P2 sequences showed the existence of putative prophage groups different from the known phages of *Vinavirales*^42^ (Data S6.2–S6.4). Five prophages integrated in the genomes of Alphaproteobacteria clustered closely with Jorvik and *Marinomonas* phage YY, whose host is likely misannotated (Data S6.2–S6.4, Table S1).

## Discussion

### Jorvik-like phages represent a new family-level group of membrane-containing circular dsDNA phages

Phage Jorvik is a unique double jelly-roll virus. This double jelly-roll classification is supported by the capsid diameter of around 59 nm, the presence of an inner membrane, the decoration of pentons with spikes (Figures 1D and 1E) and bioinformatic comparison with the archetypal *Corticovirus* phage PM2 (Figure 6; Table S2). To our knowledge, it is only the second member of the order *Vinavirales* that has been characterized in detail, after phage PM2.^1^^,^^6^^,^^7^^,^^18^ Moreover, it is the first characterized freshwater phage of the group. This does not come as a shock since our data demonstrate that established laboratory methods used for phage propagation and storage are unsuitable for work with these types of phages. Indeed, if a surreptitious lysogenic strain of phage Jorvik had not been available, the phage is likely to have been lost a long time ago. The modifications to existing phage isolation and manipulation methodologies, e.g., immediate processing of the isolation material without storage, reduced centrifugation and filtration steps, propagation of the phage via soft agar overlaying on a daily basis, long term storage of the phage in the form of frozen agar stabs, can be applied to the discovery and study of other membrane-containing dsDNA phages.

The most striking finding was that repeated rounds of centrifugation as well as filtration led to a rapid decline of the phage titer. Since centrifugation and filtration of samples is a common procedure when isolating and handling environmental phages,^43^^,^^44^ many membrane-containing dsDNA phages could easily be lost during purification. Also, due to practical considerations, environmental samples are likely to be stored for various lengths of time prior to analysis. As demonstrated here for Jorvik, storage at 4°C for even one day leads to an order of magnitude loss of titer. Thus, the few membrane-containing dsDNA phages isolated so far are likely to be a gross underestimate of their true abundance. Interestingly, even though the survival of the phage in laboratory conditions was low, the virions were quite robust against pH shifts and freeze-thawing cycles. This contrasts with phage PM2 where one cycle of freeze-thawing led to >99% loss of titer.^7^ The virions also seem to favor low salt conditions reflecting the freshwater origin of this phage.

A good estimate of the environmental abundance of phages can be inferred from the presence of homologous prophages residing in bacterial genome sequences. In the case of phage Jorvik, more than 800 similar putative prophage sequences were found throughout the Proteobacteria (Pseudomonadota) phylum mainly in aquatic isolates. These included previously characterized phages belonging to *Vinavirales*, which group outside of Jorvik-like phage cluster (Figure 5, Data S5 and S6.2), suggesting Jorvik-like phages form a separate taxon at the same level as current *Corticoviridae* and *Autolykivirdae* families. Similar observations were recently made for fNo16-like phages infecting *Vibrio* species,^21^ further suggesting that a major redefinition of taxonomy within the order *Vinavirales* is required.

The phage Jorvik2 is capable of clearing liquid cultures of *Rhodobacter* strains in several hours even when present at an MOI as low as 0.001 (Figures 2C–2E). This makes Jorvik2 highly virulent even when compared to potentially therapeutic phages,^45^^,^^46^ and thus likely to be ecologically relevant. While we corrected the MOI based on our estimate of the efficiency of plating, one needs to consider that other unknown conditions may influence the real efficiency of plating and the measured MOI might still be underestimated. Interestingly, the fast-growing *R. capsulatus* strain St. Louis was capable of escaping clearance by the phage in one biological replicate (Figure 2E). This suggests that cells entering later growth phases become less susceptible to infection, which was supported by the observation that plaque production was hampered when the phage was plated with late log-phase to stationary phase cells compared to early log-phase cells (Data S1.2). No plaque formation nor clearing of the culture was observed when cells were infected in RCV defined media or during anaerobic cultivation. In all of these conditions, *R. capsulatus* cells tend to produce more extracellular polysaccharide,^47^^,^^48^ which may hinder the phage’s ability to infect.

### Jorvik structural operon

The phage Jorvik genome is 8,762 bp, which is the smallest genome of the known membrane-containing dsDNA phages, and contains three distinct operons. The largest operon is a structural operon that is organized with equivalent synteny to other members of *Vinavirales* (Figure 6). The major protein products are P1 (receptor-binding spike), P2 (major capsid protein), P3, P7 and P8 (inner membrane protein), and P10 (unknown function). Interestingly, the putative inner membrane protein P8 was the only PM2 homologue that was not identified as part of the virion by mass spectrometry analysis. P8 is short and hydrophobic and thus hard to detect using MS methods, therefore its absence could be a false negative and its presence in the sample cannot be ruled out. Another interesting protein is a putative N-acetyltransferase designated Gp12, which is encoded between P8 and P10. The protein is conserved among the phages of *Vinavirales*, with the notable exception of phage PM2 (Figure 6).^42^ MS analysis detected only a very low amount of Gp12 in the phage-enriched sample, suggesting that it is not part of the virion but rather it is necessary for the acetylation of other structural components, similar to the function of Gp14 from *Salmonella* phage P22.^49^

Homologues of phage PM2 conserved inner membrane proteins P5 and P6 could not be identified in Jorvik phages. In the genome, they are substituted with Slt and a hypervariable region encoding several short hydrophobic proteins Gp16-19 (Table S1). Since Gp16-19 are present in a structural module and were detected in the virion-enriched sample, they could be structural virion membrane proteins. Their low sequence conservation might be a result of co-evolution with bacterial host factors, for example, they could be involved in virus-host membrane fusion. A putative holin gene, *gp20*, is located at the very end of the structural operon. A homologue of this gene product from *Marinomonas* phage YY has predicted similarity to the *R. capsulatus* gene transfer agent holin^50^ (HHpred; PF11351.11, E-value = 0.03, Score = 37.17; region 4–109 out of 118 AA).

### Jorvik replication operon

The replication operon encodes two gene products. The first is the HTH domain-containing protein Gp1, which we propose is involved in the lysis-lysogeny decision of the phage.

The Gp1 protein of virulent phage variant Jorvik2 carries a mutation in a key structural residue and a duplication of the putative upstream regulatory region. It is likely that these changes promote the lytic replication, which explains why Jorvik2 forms clear plaques and why we could not isolate a stable lysogen of this variant.

The second protein encoded in the locus is the putative replication protein Gp2. Gp2 substantially differs from the replicases of characterized phages of the *Tectiliviricetes* (Figures 6 and S2D), having only 212 amino acid residues and two distinctive domains. In comparison, both PM2 and PRD1 replicases have more than 500 residues. The N-terminal domain of Jorvik replicase (34–116 AA) is similar to the lytic replication protein of coliphage P1 (HHpred; P19654, E-value = 2.1e-5, Score = 55.9); while the C-terminal domain does not resemble any characterized proteins. Due to the short length of Gp2, we speculate that additional host factors are required for Jorvik genome replication and that the C-terminal domain might be involved in the interaction with those factors. A recent metagenomic analysis reported that the major distinguishing factor of *Vinavirales* phages is the type of replicase protein,^42^ which appears to coevolve with a specific host to allow for optimal function.

### Jorvik packaging operon

The reverse strand packaging module encodes the packaging ATPase P9, putative endolysin M15, P15-like repressor Gp7 and two hypothetical proteins, Gp4 and Gp6. The function of Gp7 is predicted from the fact that its N-terminal domain shows high similarity to known repressors including P15 of phage PM2 (HHpred; Q9XJS8, E-value = 3.8e-6, Score = 59; region 12–61 out of 149 AA) and cI repressor of phage lambda (HHpred; P03034, E-value = 4.9e-5, Score = 51; region 5–58 out of 149 AA). The C-terminal domain is rich in prolines, and its function is unknown. Upstream of this gene lies an intergenic region (sequence 3263–3448) containing a putative CtrA-binding site (3265–3276), a non-coding repeat sequence identified by blastn (3337–3384), and two promoters for expression of two oppositely facing operons (Figure 1F). In phage PM2, the P15 repressor affects expression from two oppositely facing promoters^51^ and Gp7 might work similarly. We believe CtrA, Gp7 and this intergenic region play a major regulatory role of the phage life cycle, but the exact molecular mechanism of the regulation remains to be determined.

The function of protein Gp6 may lie in virion membrane assembly. The *gp6* gene precedes the packaging ATPase ORF, which is an equivalent location to the membrane assembly factor gp-h of other *Vinavirales* phages. It also possesses a weak similarity to protein P10, the gp-h homologue in phage PRD1^42^ (Figure S2C). Moreover, the protein was abundant in the partially purified phage sample. This can be explained by the incorporation of the protein in mature virions or virion assembly intermediates. The conservation of a virion membrane assembly factor across genome packaging modules of *Tectiliviricetes* phages is striking and more research is required to explain if and how the factor is involved in the packaging process. The existence and potential function of the last hypothetical product of the operon, Gp4, remains unknown.

### Jorvik lytic enzymes

Phage Jorvik encodes two lytic enzymes. Slt (Gp15) is encoded in the structural operon while M15 (Gp3) is encoded in the packaging operon. Both Slt and M15 were identified in the phage-enriched sample by MS, with Slt being more abundant. Many phages encode two PG hydrolases, a virion-associated hydrolase and an endolysin.^52^^,^^53^ The former is required for creating a pore in the cell wall during the injection of DNA into the host. The latter is required for the release of infectious virions after their assembly. In the phages of *Vinavirales*, functional homologues of Slt in the same genome position can be identified (Figure 6).^42^ In contrast, M15 homologues were found only among Jorvik-like phages infecting Alphaproteobacteria and as such are unique. Based on the genome position and MS data, we hypothesize Slt is a virion-associated hydrolase while M15 is an endolysin, analogous to the P7 and P15 hydrolases of phage PRD1.^54^

The isolation and detailed characterization of phage Jorvik prove that membrane-containing dsDNA phages of *Vinavirales* are widespread predators of aquatic niches. Modifications to classical laboratory methodologies and approaches described here can be applied in the cultivation of other membrane-containing dsDNA bacterial viruses and may drive a revolution in their study.

### Limitations of the study

The instability of the phage in laboratory conditions might have affected the outcomes of some microbiological assays such as phage growth curve and burst size determination, leading to an underestimation of the real values. Also, due to this instability only a partially purified sample of the phage could be analyzed by mass spectrometry, thus the virion association of individual components needs to be interpreted carefully. The study did not target the molecular mechanisms of phage integration and operon regulation, the mechanisms proposed were based on similarity with homologous proteins and the behavior of mutant phenotypes. Due to very low sequence similarity among proteins of Tectiliviricetes, the support of several phylogenetic tree branches is low. For this reason, we did not provide any extensive interpretation of the topology of trees.

## STAR★Methods

### Key resources table

**Table:** 

REAGENT or RESOURCE	SOURCE	IDENTIFIER
**Bacterial and virus strains**		

See Tables 1 and S3 for a complete list of *Rhodobacter* strains used in this study.	N/A	N/A
E. coli S17-1	The Leibniz Institute DSMZ; www.dsmz.de	DSM 9079
*E. coli* HST08	Takara Bio, www.takarabio.com	Cat.#636763
*E. coli* Rosetta (DE3) pLysS	Novagen, www.sigmaaldrich.com	Cat.#71403-M
E. coli NEB 10-beta Competent	New England Biolabs	Cat#C3019

**Chemicals, peptides, and recombinant proteins**		

Chloroform	Merck Life Science Limited	Cat#132950-1L
RNase A	VWR International	Cat#A2760.0100
Q5 DNA polymerase	New England Biolabs	Cat#M0491L
SafeBLUE stain	NBS Biologicals	Cat#NBS-SB1L
Dnase I		
PEG8000	Promega	Cat#V3011
EcoRV	New England Biolabs	Cat#R0195S

**Critical commercial assays**		

NEBuilder Cloning Kit	New England Biolabs	Cat#E5520

**Deposited data**		

Rhodobacter phage Jorvik – genome sequence	This paper	Genbank: OP588643
Marinomonas phage YY – third party annotation	This paper	Genbank: BK062760
Rhodobacter capsulatus B10 – whole genome shotgun	This paper	Genbank: JAOTPJ010000000
Mass spectrometry data of partially purified Jorvik lysate	This paper	MassIVE: MSV000090713

**Oligonucleotides**		

See Table S5 for all primers used in this study.	N/A	N/A

**Recombinant DNA**		

ZeroBlunt Topo vector	Thermo Fisher Scientific	https://www.thermofisher.com/uk/en/home/life-science/cloning/topo/high-fidelity-blunt-end-topo-cloning.html
pCM66T plasmid	Fogg^34^	https://www.addgene.org/74738/
pETYSBLIC3c	Fogg and Wilkinson^55^	N/A

**Software and algorithms**		

Prokka 1.14.5	Seemann^56^	https://bioweb.pasteur.fr/packages/pack@prokka@1.14.5
GeneMarkS 4.28	Besemer et al.^57^	http://exon.gatech.edu/genemark/genemarks.cgi
Artemis	Carver et al.^58^	https://www.sanger.ac.uk/tool/artemis/
Basic Local Alignment Search Tool	Altschul et al.^59^	https://blast.ncbi.nlm.nih.gov/Blast.cgi
HHpred	Zimmermann et al.^41^	https://toolkit.tuebingen.mpg.de/tools/hhpred
AlphaFold2	Jumper et al.,^60^ Mirdita et al.^61^	https://colab.research.google.com/github/sokrypton/ColabFold/blob/main/AlphaFold2.ipynb
DALI search	Holm^62^	http://ekhidna2.biocenter.helsinki.fi/dali/
Genome2D web tool	University of Groningen	http://genome2d.molgenrug.nl/g2d_pepper_transterm.php
Entrez	NCBI	https://www.ncbi.nlm.nih.gov/sites/batchentrez
NCBI e-utilities	Kans^63^	https://www.ncbi.nlm.nih.gov/books/NBK25501/
NCBI tax identifier	NCBI	https://www.ncbi.nlm.nih.gov/Taxonomy/TaxIdentifier/tax_identifier.cgi
regular expression search tool	Regex101	https://regex101.com/
MAFFT v7.487 e-ins-I algorithm	Katoh and Standley^64^	https://mafft.cbrc.jp/alignment/software/
trimAl v1.4 algorithm automated1	Capella-Gutiérrez et al.^65^	http://trimal.cgenomics.org/trimal
iqtree2 v2.0.6	Minh et al.^66^	https://github.com/iqtree/iqtree2
FigTree v.1.4.4.	University of Edinburgh	http://tree.bio.ed.ac.uk/software/figtree/
VICTOR web server	Meier-Kolthoff and Göker^40^	https://ggdc.dsmz.de/victor.php
IMOD 4.11.20 Tomography package Etomo	Kremer et al.^67^	https://bio3d.colorado.edu/imod/
UCSF ChimeraX	Goddard et al.^68^	https://www.cgl.ucsf.edu/chimerax/
ggplot2 system of the package R	Wickham^69^	https://www.r-project.org/
OrthoANIu web tool	Lee et al.^70^	https://www.ezbiocloud.net/tools/orthoaniu

**Other**		

NuPAGE 10% BisTris gel	Invitrogen	Cat#NP0301BOX
50 cm EN C18 PepMap column	Thermo Fisher Scientific	Cat#164560
200 mesh R2/1 copper Quantifoil® grids	Agar Scientific	Cat#AGS174-1-100
HisTrap 5ml FF column	Cytiva	Cat#17525501
Super Ni-NTA resin	Generon	Cat#NB-45-00042-10
6 nm colloid gold fiducials	Aurion	Cat#406.011

### Resource availability

#### Lead contact

Further information and requests for resources and reagents should be directed to and will be fulfilled by the lead contact, Paul Fogg (paul.fogg@york.ac.uk).

#### Materials availability

All unique reagents or materials generated in this study will be made available on request by the lead contact, but we may require a completed materials transfer agreement if there is potential for commercial application.

### Experimental model and subject details

#### Bacterial strains

The bacterial strains used in this study are summarized in Tables 1 and S3. In addition, several *Escherichia coli* strains used for cloning are specified in the description of the corresponding methods. *Rhodobacter* strains were grown either in minimal media RCV^71^ or rich media YPS,^26^ *E. coli* strains were grown in LB. *Rhodobacter* strains complemented for the production of CtrA were cultivated in the presence of 10 μg.ml^-1^ of kanamycin. Unless otherwise stated, *Rhodobacter* strains were incubated aerobically at 30°C and *E. coli* strains at 37°C. For anaerobic cultivation, bacteria were incubated in sealed 15 ml screw-cap glass tubes filled with YPS up to the rim or YPS plates in Anaerocult® A jars (Merck Millipore). The samples were incubated at 30°C in an illumination cabinet, placed 30 cm from three 40 W light-emitting tubes (Panasonic FL40SS・W/37c).

#### Bacteriophage Jorvik

The phage was propagated using YPS soft agar overlaying. The overnight culture of the propagation strain was mixed with phage lysate, then 3 ml of soft agar (YPS + 0.7% w/v agar) were added and overlaid on a YPS agar (1.5% w/v) plate. The plate was incubated at 30°C overnight. The soft agar containing phage particles was harvested using a bacterial loop and resuspended in YPS media. The tube was vortexed vigorously and centrifuged at 6000 RCF for 4 min to separate the agar from the phage solution. Repeated or high-speed centrifugation was avoided as we observed that these procedures led to substantial loss of plaque forming units. Filtration of phage lysates was not performed as this also led to a large reduction of phage titre; instead, the supernatant was transferred to a clean tube using a 30G gauge needle, stored in the fridge and used for any experiments within 8 h. For pH and salt stability assays, soft agar containing the phages was resuspended in 5-10 times of excess of YPS by passing through a 30G gauge needle and directly used for experiments. This led to one order of magnitude higher titres, but with the presence of residual agar and cell debris in a lysate.

Unless otherwise stated, the phage titre was estimated by a drop assay. Here, 10 μl of diluted samples were dropped onto freshly solidified soft agar containing early log phase culture of *Rhodobacter capsulatus* YW1 (OD_600nm_ = 0.1-0.25) and left with an open cover for 7-10 minutes in a flow hood to dry. For estimating the titre from soft agar overlay, the host cell culture was mixed with diluted phage samples before the addition of soft agar and subsequently plated. For the estimation of efficiency of plating for bacterial cultures at different growth phases, OD_600nm_ of log and stationary phase cultures was measured, cells diluted to OD_600nm_=0.10 in YPS and 250 μl of the culture was used for phage plating either by soft agar overlay or drop assay as stated.

### Method details

#### Standard laboratory handling stability assay

For the chloroform treatment, 5 % v/v of chloroform were added to the lysate. The sample was vortexed, incubated for 10 minutes on a bench, centrifuged at 6000 RCF for 4 min, and 100 ul of the supernatant from the top of the tube was transferred to a new tube and titred. For the phage filtering, Merck Millex®GP PES Membrane 0.22 μm (Merck Millipore), Minisart NML Plus cellulose acetate membrane 0.45 μm (Sartorius), yellow syringe filters PES membrane 0.45 μm (Starlab), Nylon 66 membrane 0.2 μm (Supelco) and Durapore® PVDF membrane 0.22 μm (Merck Millipore) filters were used to filter 2 ml of phage lysate. For centrifugation experiments, 500 ul of phage lysate were spun at 6000 RCF for 4 min, and 100 ul of the supernatant from the top of the tube were subsequently transferred to a fresh tube and titred.

#### Isolation of lysogenic strain

Phage lysate was mixed with the *Rhodobacter capsulatus* St. Louis overnight culture and plated using a soft agar overlay. After the overnight incubation, several plaques were picked with a sterile tip and streaked onto fresh plates and incubated for two days to get individual phage-resistant colonies. Single colonies were restreaked onto a fresh plate and subsequently inoculated into RCV. After two days of incubation in RCV, the cells were spun, and the supernatant was dropped onto St. Louis WT strain to discover phage-inducing lysogens. Colonies positive for phage production were subsequently tested for the presence of the phage genes *gp15* (*slt)* and *gp3* (*M15)* by PCR. To verify the colonies do not come from the parental B10 strain, after obtaining the lysogen sequence, the average nucleotide identity between St. Louis C1, B10 and St. Louis WT strains was calculated using OrthoANIu web tool.^70^ For St. Louis C1 and B10, contig sequences obtained from Illumina sequencing were used as an input. For St. Louis WT, contig sequences deposited under GenBank project NZ_VIBE00000000.1 were used. This resulted in 99.95 and 99.47 % average nucleotide identity when St. Louis C1 was compared to St. Louis WT and B10 respectively.

#### Growth characteristics

The growth of different strains in presence of phage was determined using OD_600nm_ measurement on Spectrostar Nano Microplate Reader (BMG LabTech). The strains were grown to OD_600nm_ 0.2 to 0.5 and diluted to a final OD_600nm_=0.1 in 200 μl of media containing between 2 and 7 × 10^4^ phage particles per well (Data S1.5). The plate was incubated at 30°C, and the OD_600nm_ was measured at 30 min intervals for 20 h. The plates were shaken orbitally at 100 rpm for 10 s before each measurement. The range of phage titre rather than specific titre value was used for growth experiments because of the inherent laboratory instability, a fresh phage lysate was used for each experiment without previous knowledge of its precise titre.

The growth curve of phage was determined using an approach similar to Kropinski, 2018.^72^ YW1 strain was used as a host. Log phase cultures (OD_600nm_ = 0.2-0.5) diluted to OD_600nm_ = 0.1 and 2 × 10^4^ to 7 × 10^4^ phages were added to 3 ml of final culture volume. The mixture was then incubated at 30°C and gently vortexed before sampling each time point. Ten and hundred-fold dilutions of the infected cells were prepared one hour post-infection, which was still in the lag phase of the phage. For sampling, 200 μl aliquots were spun at 12 000 RCF for 2 min and plated with log-phase YW1 strain using soft agar overlaying. Plaques were counted the next day. The burst size was calculated as the difference between the averages of plaque-forming units before and after the lytic burst.

For the adsorption assay, an approach similar to Kropinski, 2009^36^ was employed. In brief, 100 μl of between 1 × 10^5^ and 9 × 10^5^ phage was added to 900 μl of the log-phase grown culture, with a final OD_600nm_ of 0.1. The mixture was incubated at 30°C, shaking at 90 rpm. The aliquots were diluted 20 times in ice-cold YPS, spun at 12 000 RCF for 2 min and 200 μl were plated with a 100 μl of log-phase YW1 culture using the soft agar overlay. Plaques were counted the next day. To estimate the adsorption rate, the equation from Kropinski, 2009^36^ was used.

#### Stability characteristics

For the heat stability assay, 250 μl of phage lysate was incubated in a heat block set at a specific temperature for 30 min. The titre of the phage was then estimated using a soft agar drop assay. For the freeze-thawing experiment, 250 μl of phage lysate with the titre between 1 × 10^5^ and 1 × 10^7^ PFU.ml^-1^ was frozen at -80°C for 30 min and then thawed at room temperature for another 30 min. After the last thawing cycle, the phage titre of all samples was estimated by a soft agar drop assay. For the pH and salinity experiment, YPS media was modified to different salt concentrations and pH by the addition of NaCl and HCl/NaOH respectively. Precise pH was determined using pH electrode InLab Easy BNC (Mettler Toledo) and adjusted as necessary. The phage lysate with the titre between 1 × 10^5^ and 1 × 10^7^ PFU.ml^-1^ was diluted 1:19 with the modified YPS media and incubated for 1.5 h at room temperature. After plating the phage, the lysates were transferred to the fridge and the titre was estimated again after an additional 22.5 h (24 h in total) of incubation in the modified YPS media. All the growth and stability experiments were done in three independent biological replicates, utilising different overnight cultures and different phage lysates.

#### DNA isolation and sequencing

The genome of Jorvik1 was isolated from 18 ml of lysate with a titre of 1 × 10^7^ PFU.ml^-1^. The phage was pelleted by the addition of PEG8000 to a final concentration of 10% (w/v), incubation at room temperature for 10 min and centrifugation at 10 000 RCF for 10 min. The supernatant was discarded, and the pellet was resuspended in 500 μl phage buffer (50 mM NaCl, 10 mM Tris pH7, 10 mM MgSO_4_). Cellular nucleic acids were removed by digestion with 0.2 mg.ml^-1^ DNaseI and 0.02 mg.ml^-1^ RnaseA and incubation at 37°C for 1 h. EDTA was added to a final concentration of 50 mM, then encapsidated DNA was extracted using the “Purification of Nucleic Acids by Extraction with Phenol:Chloroform protocol”.^73^ Purified DNA was linearized with blunt ends using EcoRV (New England Biolabs) and inserted into the ZeroBlunt Topo vector (Thermo Fisher Scientific). The genome of Jorvik2 was isolated from 6 ml of a lysate with the titre 4 × 10^6^ in a similar manner. The integrity and amount were sufficient for direct sequencing. The genomic DNA of strains B10 and St. Louis C1 were isolated from 1.5 ml of stationary aerobic culture grown in YPS according to the published manual.^73^ All the samples were sequenced using Illumina sequencers (HiSeq/NovaSeq) and a 250 bp paired-end protocol. Genome sequencing was provided by MicrobesNG (http://www.microbesng.com).

#### Estimation of phage integration site in St. Louis C1

The raw reads of St. Louis C1 were searched for the integration site by filtering for reads containing the first and last 17 nt of the NODE_41 contig sequence, which contained the circular sequence of the phage, using the “grep” command. This resulted in 596 reads, including the reverse complements. The sequence upstream to the start of the NODE_14 contig sequence and downstream to the end of the sequence was analysed in Vim text editor, resulting in two populations, one (479 reads) corresponding to the phage circular sequence and the other (91 reads) corresponding to the bacterial integration site in *cbbII* operon. The upstream/downstream sequence of the remaining 26 reads was shorter than 6 nt and these were not assessed.

#### *R. capsulatus* genes knockouts

Knockouts in *R. capsulatus* were created by gene transfer agent mediated gene replacement.^34^ pCM66T plasmid constructs were created with a gentamicin resistance cassette flanked by 500-1000 bp of DNA from either side of the target gene (Table S5). Assembly was achieved by a one-step, four-component NEBuilder reaction and transformation into NEB 10-beta cells (New England Biolabs). All oligonucleotides were obtained from IDT and designed with an optimal annealing temperature of 60°C when used with Q5 DNA Polymerase (New England Biolabs). All cloning reactions were carried out with NEBuilder according to the manufacturer’s guidelines (New England Biolabs).

Deletion constructs were introduced into the *E. coli* conjugation strain S17-1 and then transferred to the *R. capsulatus* gene transfer agent hyperproducer strain DE442 by solid phase conjugal mating. One millilitre aliquots of overnight cultures of the *E. coli* S17-1 donor and *Rhodobacter* recipient strains were centrifuged at 5000 RCF for 1 min, washed with 1 ml YPS medium, centrifuged again and resuspended in 200 μl YPS. Ten microlitres of concentrated donor and recipient cells were mixed and spotted onto YPS agar or spotted individually as negative controls. Plates were incubated overnight at 30°C. Spots were scraped, suspended in 100 μl YPS broth and plated on YPS + 100 μg ml^-1^ rifampicin (counter-selection against *E. coli*) + 10 μg ml^-1^ kanamycin (plasmid selection). Plates were incubated overnight at 30°C then restreaked onto fresh agar to obtain single colonies.

A standard gene transfer agent bio-assay^74^ was carried out to replace the intact chromosomal gene with the deleted version using 10 μg.ml^-1^ gentamicin for selection. Successful knockouts were confirmed by Sanger sequencing (Eurofins Genomics).

#### Bioinformatics and data visualization

ORFs were identified using *ab initio* prediction of Prokka 1.14.5^56^ and GeneMarkS 4.28^57^ with the “phage” algorithm. The annotation and circular maps were done in Artemis.^58^ The predicted function of protein was based on primary sequence similarity using blastp,^59^ the comparison of profile hidden Markov models using HHpred^41^ and predicted tertiary structure similarity using a combination of AlphaFold2^60^^,^^61^ and DALI search.^62^ The terminators were predicted using the Genome2D web tool [http://genome2d.molgenrug.nl/g2d_pepper_transterm.php].

Psi-blast search against bacterial and viral taxids using matrix BLOSUM=45 was performed using the less conserved major capsid protein P2 sequence until convergence.^59^^,^^63^ Then, a list of nucleotide seqIDs encoding proteins found in all iterations was determined using NCBI batch Entrez search against identical protein groups database [https://www.ncbi.nlm.nih.gov/sites/batchentrez]. Unique Gis encoded by the longest nucleotide sequence were filtered and a list of proteins encoded within the sequences was generated using NCBI e-utilities.^63^ This list served as a subject for psi-blast with the packaging ATPase P9 until convergence using matrix BLOSUM=45. The position in the nucleotide sequence of these new hits was retrieved using a batch Entrez search and compared to the original major capsid protein P2 hits. Hits originating from the same nucleotide sequence with less than 10000 nt from each other were filtered using awk script and considered putative prophage sequences. The taxonomy information of the hits was retrieved using the NCBI tax identifier [https://www.ncbi.nlm.nih.gov/Taxonomy/TaxIdentifier/tax_identifier.cgi]. The putative CtrA-binding site and *dif* motifs were identified using consensus sequences and regular expression search tool [https://regex101.com/].

#### Phylogeny analysis

Phylogeny analysis was performed according to Grybchuk et al*.*^75^ In brief, a list of homologous proteins to those from Jorvik was generated by PSI-BLAST. The search was performed using the matrix BLOSUM=45 and the default settings until converging. The taxonomy database used for the search was *Tectiliviricetes* in the case of the major capsid protein P2, Alpha-proteobacteria (filtered out after the convergence) and *Tectiliviricetes* in the case of the packaging ATPase P9. The complete sequences were then aligned using MAFFT v7.487 e-ins-I algorithm.^64^ The uninformative columns were cropped using trimAl v1.4 algorithm automated1^65^ and the phylogenetic tree was computed by maximum likelihood method using software iqtree2 v2.0.6^66^ with a bootstrap test. A similar approach was performed using P9 and P2 sequences from Data S6.1 as an input, with the final tree computed using fast bootstrap test. The trees were visualized using FigTree v.1.4.4. [http://tree.bio.ed.ac.uk/software/figtree/].

For the Genome-BLAST Distance Phylogeny, genomic sequences classified to *Autolykiviridae*, *Corticoviridae* were retrieved from GenBank and together with the sequence of Jorvik used as a nucleotide input to VICTOR web server [https://ggdc.dsmz.de/victor.php].^40^ The phylogeny was performed on a predicted amino acid sequence under settings recommended for prokaryotic viruses, using a recommended greed-with-trimming algorithm of formula D6.

#### Proteomic analysis

The phage sample for proteomic analysis was prepared in the same way as for the genome isolation. After pelleting and resuspending in B buffer, extra polishing was performed. Here, most remaining bacteria were separated by 6 000 RCF/4 min/8°C spin. Phage particle aggregates were enriched from the supernatant by an additional 17 000 RCF/25 min/8°C spin, resuspended in B buffer to final A_280nm_ ∼ 2 and used for LC-MS/MS analysis performed by The Centre of Excellence in Mass Spectrometry, University of York. Here, the phage-enriched sample was run 1 cm into a NuPAGE 10% BisTris gel (Invitrogen) and stained with SafeBLUE stain (NBS Biologicals). The stained gel segment was excised, destained, reduced with DTE and alkylated with iodoacetamide. Protein was in-gel digested using Promega sequencing grade trypsin and incubation overnight at 37°C. The resulting peptides were analysed by LC-MS/MS over a 1 h acquisition with elution from a 50 cm EN C18 PepMap column (Thermo Fisher Scientific) driven by an mClass UPLC (Waters) onto an Orbitrap Fusion Tribrid mass spectrometer (Thermo Fisher Scientific) operated in DDA mode. MS1 spectra were acquired at high resolution in the Orbitrap mass analyser. MS2 spectra were acquired in TopSpeed mode in the linear ion trap after HCD fragmentation.

LC-MS data in .raw format were imported into Progenesis QI for peak picking with tandem mass lists exported in .mgf format for database searching using Mascot. Data were searched against the combined Jorvik and NCBI *R. capsulatus* YW1 databases appended with common proteomic contaminants. Peptide matches were filtered to 1% FDR using the Percolator algorithm and as determined empirically by comparison to a reverse database search. Accepted peptide identifications were imported into QI and associated with the LC-MS chromatographic data. Results were further filtered to require a minimum of two unique peptides per protein identification. As a metric for relative abundance between proteins the Top3 approach has been used, whereby the best three responding peptide precursor ion areas for each protein are extracted and compared.

#### Electron microscopy

For cryo-EM grids, a confluently lysed plate of phage was washed with 2 ml of RCV using a pipette and the media was spun at 4000 RCF for 6 min. The pellet, containing aggregates of virus particles as well as particles attached on host membranes, was resuspended in the residual amount of liquid, mixed with 6 nm colloid gold fiducials (Auriol), applied onto glow-discharged 200 mesh copper Quantifoil® grids and plunge frozen in liquid ethane. The grids were imaged using Glacios TEM (Thermo Fisher Scientific) operated at 200 kV at the cryo-EM facility of the University of York.

#### Tomography data collection and reconstruction

Cryo-electron tomography data were acquired at ×73 000 magnification and -5 μm defocus using -60,60° dose symmetric tilt series acquisition with 3° increment and a total dose of 2.9 e^-.^Å^-2^ per each tilt image. The tilt images were aligned using fiducial alignment in IMOD 4.11.20 Tomography package Etomo.^67^ Subsequently, the tilt series were 6 times binned, reconstructed into 3D tomograms and filtered using the non-linear anisotropic diffusion algorithm of Etomo.

#### Production of the lytic enzymes

The *M15* and *slt* genes were cloned using HiFi assembly (NEB) of PCR-generated products with pETYSBLIC3c plasmid.^55^ The primers used for amplification are summarised in Table S5. The assembled plasmids were transformed into *E. coli* Stellar cells (Takara Bio) and individual colonies were verified for the presence of the insert by sequencing (Eurofins Genomics GATC). The plasmids were subsequently isolated from verified colonies and transformed to expression strain *E. coli* Rosetta (DE3) pLysS (Novagen). The expression was induced with 0.1 mM IPTG at 25°C (M15) and 20°C (Slt) respectively. The pellets were resuspended in buffer B_M15 (Tris 20 mM pH=7.5, NaCl 100 mM, CaCl_2_=5mM, Imidazole = 20 mM) and B_Slt (Tris 20 mM pH=8.5, NaCl 400 mM, CaCl_2_=5mM, Imidazole = 10 mM), sonicated using Sonoplus HD2070 (Bandelin) and purified using HisTrap 5ml FF column (GE Healthcare) and step imidazole gradient. The fraction containing proteins was pooled, buffer exchanged until reaching the original imidazole concentration and the his-tag was cleaved overnight in presence of 1mM DTT and 20 μg of 3C protease. His tag and the protease were removed from the protein samples by 20 min incubation with Super Ni-NTA resin (Generon).

#### Zymogram and the plate lysis assay

Zymogram and plate lysis assays were performed according to Benesik et al.*, 2018*.^52^ In brief, the analysed proteins were diluted in B_M15 or B_Slt buffer to a concentration of 0.6 mg.ml^-1^, based on Bradford protein assay calibrated on BSA standard. For the zymogram, protein samples were loaded into SDS-PAGE gels, containing 15 % acrylamide and 200 μl of *R. capsulatus* SB1003 crude peptidoglycan, prepared according to Fogg et al.*, 2012*.^76^ The gel ran at 150 V for 75 minutes, was washed three times for 15 minutes in fresh distilled water, placed in a fresh container with distilled water and incubated at 30°C/40 rpm for 3 hours. The gel was then stained with a solution of 0.1% Methylene Blue and 0.001 M KOH for 3 hours and destained in distilled water. For the plate lysis assay, 500 μl of *R. capsulatus* SB1003 crude peptidoglycan were incorporated into 4 ml of 0.4% (w/v) dH_2_O agar and poured on a clean petri dish. Protein samples were then dropped onto the plate and incubated for 3-12 hours at 30°C until clear zones of peptidoglycan degradation appeared.

### Quantification and statistical analysis

The structural figures were created using UCSF ChimeraX (*67*). All the charts were plotted using the ggplot2 system (*68*) of the package R [https://www.r-project.org/].

## Data Availability

• Nucleotide sequence data reported are available in DDBJ/ENA/GenBank: OP588643 for Rhodobacter phage Jorvik, the Third Party Annotation Section of the DDBJ/ENA/GenBank: BK062760 for Marinomonas phage YY. Rhodobacter capsulatus B10 strain Whole Genome Shotgun project has been deposited at DDBJ/ENA/GenBank: JAOTPJ000000000. The version described in this paper is version JAOTPJ010000000. Raw LC-MS/MS data of partially purified Jorvik lysate are deposited at MassIVE: MSV000090713. All datasets are publicly available as of the date of publication. All AlphaFold2-predicted models which are discussed within the manuscript are attached as Data S7. • This paper does not report original code. • Any additional information required to reanalyse the data reported in this paper is available from the lead contact upon request.

## Supplemental information

Document S1. Figures S1–S3 and Tables S1–S5

Data S1. Raw data of microbiological assays, related to Figures 2 and 3 S1.1) Estimation of phage Jorvik stability during incubation in media with a different pH. S1.2) Phage Jorvik stability during common laboratory manipulations. S1.3) Raw data related to the phage adsorption assay. S1.4) Raw data related to the phage growth curve. S1.5) Raw data related to *R. capsulatus* growth curve with and without the addition of phage Jorvik. S1.6) Estimation of phage Jorvik stability during heating. S1.7) Estimation of phage Jorvik stability during freeze-thawing. S1.8) Estimation of phage Jorvik stability during incubation in media with a different salt concentration.

Data S2. Identified sequences of putative gene products from Jorvik-like prophages, which are not annotated in GenBank, related to Figure 1 and Table S1

Data S3. Images of gels related to Figure 4 S3.1) Colony PCR amplification of Jorvik genes M15 and slt performed on Rhodobacter strains. a) Demonstration of the presence of the annotated genes in Rhodobacter capsulatus strain St. Louis C1. b-c) Verification of slt gene presence in 4/4 individual colonies of R. capsulatus B10 strain and 10/10 individual colonies of St. Louis C1. d) Verification that both slt and M15 are present in two individual St. Louis C1 colonies. e-f) PCR amplification of M15 (e) and slt (f) from a bank of Rhodobacter strains. Ladders 100bp and 1kb+ are from New England Biolabs. S3.2) Images of gels related to the zymogram experiment. a) Slt sample is composed of two bands as a result of an ineffective cleavage of the his-tag by 3C protease. The red arrow highlights the uncleaved product and the gray arrow the cleaved product. The expected molecular weights of the product are shown in numbers. Gel lanes descriptions: M, marker PageRulerTM 10–250 kDa (Thermo Fisher Scientific); 1, metal affinity purified Slt with the sample loaded in higher (1a) and lower (1b) concentration; 2, purified Slt with 3C protease after overnight incubation; 3, pellet bead fraction of purified Slt with 3C protease; 4, a supernatant fraction of purified Slt with 3C protease with the sample loaded in higher (4a) and lower (4b) concentration. b) The complete image of the zymogram from which insets are shown in Figure 4.

Data S4. MS analysis of partially purified Jorvik2 virions, related to Figure 1 The proteins are ordered according to their abundance estimated from the area of the top three peptides identified for the protein. ID Filter = Mascot Percolator to 1% FDR and QI min 2 peptides. Phage proteins are highlighted in gray.

Data S5. Phylogenetic trees of phage Jorvik based on conserved protein sequences, related to Figure 5 S5.1) Phylogenetic trees of phage Jorvik based on the sequence of major capsid protein P9. Maximum likelihood tree based on PSI-BLAST searches in the taxid “viruses”. The confidence estimates of branches are shown next to nodes, with values > 90% shown in cyan. The low confidence separation of branches near the tree center of the tree is enclosed in blue oval. The color of the name depicts approved or officially proposed family ranks – Autolykiviridae (teal), Jorvik-like (magenta), Corticoviridae (green), fNo16-like (orange), Tectiviridae (blue), Turriviridae (gold) and unassigned (gray). The scale bar defines the branch length as the number of substitutions per site. All the used sequences for computing the tree are present in Data S5.2. S5.2) List of sequences used for building the phylogeny trees of Jorvik based on the packaging ATPase P9. S5.3) Phylogenetic trees of phage Jorvik based on the sequence of major capsid protein P2. Maximum likelihood tree based on PSI-BLAST searches in the taxid “viruses”. The confidence estimates of branches are shown next to nodes, with values > 90% shown in cyan. The low confidence separation of branches near the tree center of the tree is enclosed in blue oval. Jorvik-like phages are highlighted in bold. The color of the name depicts approved or officially proposed family ranks – Autolykiviridae (teal), Jorvik-like (magenta), Corticoviridae (green), fNo16-like (orange) and unassigned (gray). The scale bar defines the branch length as the number of substitutions per site. All the used sequences for computing the tree are present in Data S5.4. S5.4) List of sequences used for building the phylogeny trees of Jorvik based on the major capsid protein P2.

Data S6. Analysis of sequences from nucleotide databases which encode homologs of Jorvik proteins P2 and P9 in proximity, related to Figure 5 S6.1) List of sequences from nucleotide databases which encode homologs of Jorvik proteins P2 and P9 in close proximity. S6.2) Clustering of conserved proteins of putative prophages similar to Jorvik. a-b) unrooted radial trees of P9 protein (a) and P2 protein (b) based on putative prophage sequences from Data S6.1. Positions of characterized phages are highlighted. The individual tree files are attached in Data S6.3 for P9 and Data S6.4 for P2 respectively. S6.3) Raw tree file of P9 sequences, plotted in Data S6.2a. S6.4) Raw tree file of P2 sequences, plotted in Data S6.2b.

Data S7. Files of AlphaFold2-generated models discussed in the manuscript with plots of their predicted local distance difference test (pLDDT) per residue, related to Figures 1 and S2
