# Supplementary material for: Jorvik: A membrane-containing phage that will likely found a new family within Vinavirales
Source: iScience. 2023 Sep 29;26(11):108104. doi: 10.1016/j.isci.2023.108104 (PMC10589892; doi:10.1016/j.isci.2023.108104)
Supplement: Document S1. Figures S1–S3 and Tables S1–S5 [file mmc1.pdf]

iScience, Volume *26*

Supplemental information

Jorvik: A membrane-containing phage that will likely found a new family within Vinavirales

Pavol Bárdy, Conor I.W. MacDonald, Roman Pantůček, Alfred A. Antson, and Paul C.M. Fogg

A

T [°C]	Plaque formation		
18	-	+	+
20	++	++	++
22	++	++	++
28.5	++	++	++
34	++	++	++
37	-	-	-

+, impaired plaques; ++ normal plaques;
-, resistant

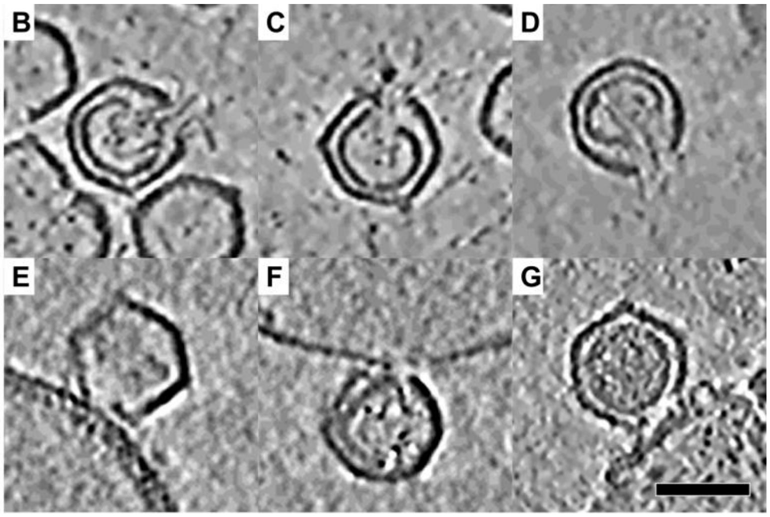

Figure S1. Characterization of plaque-forming and virion properties of phage Jorvik, related to Figure 1. A) Outcome of different incubation temperatures on phage Jorvik plaque formation. B-D) Tomograms of phage particles which show ruptured virions, with the genome escaping from the particles. E-G) Tomograms of phage particles attached to a membrane. The density of the capsid shell seems to be missing at the point of the attachment. The scale bar represents 50 nm.

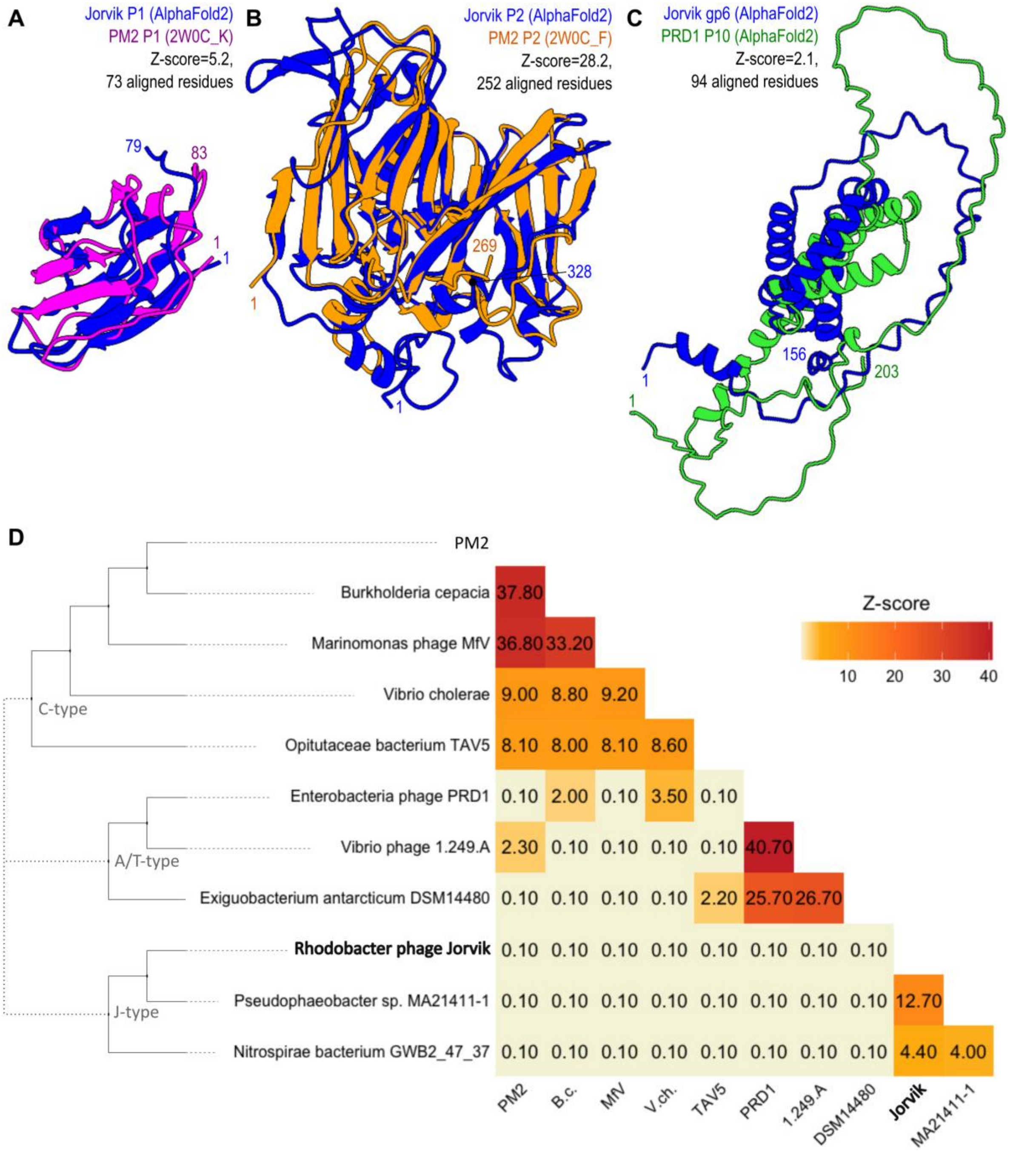

Figure S2. Characterisation of phage Jorvik by a protein structure prediction using AlphaFold2 [S1], related to Figure 1. A-C) Superimposition of Jorvik penton domain of head spike protein P1 (A), major capsid protein P2 (B) and putative membrane assembly factor gp6 (C). Z-score values of pairwise comparison as estimated using DALI server [S2] are shown. Values of a Z-score higher than 2 suggest a structural similarity. D) Clustering of the replicase protein of *Tectiliviricetes* based on the predicted models. The structural similarity is estimated by the Z-score of the all-against-all comparison made with the DALI server. Phage Jorvik is highlighted in bold. The type of replicase is named according to *Corticoviridae* (C), *Autolykiviridae/Tectiviridae* (A/T) and Jorvik (J).

```
A       Rhodobacter sphaeroides [S3]
                        GAGTCGGATAATCTGTATTATGTATTCT

        Escherichia coli [S4]
                        GGTGCGCATAATGTATATTATGTTAAAT

        Rhodobacter phage Jorvik [OP588643]
        Rhodobacter capsulatus SB1003 [NC_014034.1]
        attP 8760       GAGTCCGATAATCAGTATTTAGGGGGAG 25
        attB 1983982    AAATCCTATAATCAGTATTATGTAAAAT 1983955

        Marinomonas phage YY [MH105080.1]
        attP 164        TTATCCTATAATTGGTATTACGGCAAAA 137

        Marinosulfonomonas sp. PRT-SC04 [JPUR01000104.1]
        Left 12102      TTGGCGCATAATTAGTACTATGCTATTG 12083
        Right 2856      AAGGCGCATAATTAGTATTATGTTAAAT 2829
        attP            AAGGCGCATAATTAGTACTATGCTATTG
        attB            TTGGCGCATAATTAGTATTATGTTAAAT

        Pseudophaeobacter sp. MA21411-1 [JAJQRG010000003.1]
        Left   440011   AAAACTCATAATCAGTAGTATGGTCTGG 440038
        Right 448592    TAGTCGCATAATCAGTATTATGGTAAAT 448619
        attP            TAGTCGCATAATCAGTAGTATGGTCTGG
        attB            AAAACTCATAATCAGTATTATGGTAAAT

        Rhodobacteraceae bacterium isolate GLR160 [WTAL01000162.1]
        Left   25757    AAGGCGCATAATTGATAGTATGTTACTG 25730
        Right 16717     ACGGCGCATAATAGATATTATGTTACTT 16690
        attP            ACGGCGCATAATAGATAGTATGTTACTG
        attB            AAGGCGCATAATTGATATTATGTTACTT

        Tropicibacter sp. LMIT003 [RCTZ02000001]
        Left   83       AAGACTTATAATCAGTATTTCGGGGGCT 110
        Right 8692      AAGGCGCATAATCAGTATTATGTAACTA 8719
        attP            AAGGCGCATAATCAGTATTTCGGGGGCT
        attB            AAGACTTATAATCAGTATTATGTAACTA

        Neotabrizicola shimadae strain N10 [CP069370.1]
        Left   1429163  TTATCCGATAATCAATATTATCCCTCGG 1429136
        Right 1419826   AAATCTGATAATCAATATTATGTCATGC 1419799
        attP            AAATCTGATAATCAATATTATCCCTCGG
        attB            TTATCCGATAATCAATATTATGTCATGC

B       Jorvik [OP588643]           3265    TTAACCTTCGATTTA 3276
        R. capsulatus gafA [S5]             TTAACCAAGGGGTAA
        R. capsulatus ctrA [S5]             GTAACTACGTGTTAA
        R. sphaeroides cons. [S6]           TTAACCNTTCGTTAA
```

Figure S3. Putative motifs were discovered in Jorvik-like phages using regular expression search, related to Figure 1. A) *attP* and *attB* integration sequences of Jorvik-like phages show similarity to XerCD recombinase *dif* motifs. The canonical *dif* AT repeat motif is highlighted in cyan, the phage strand in magenta, and the host genome in yellow. The matching *att* recombination region is shown in bold, with the single mismatch shown in green. GenBank accession or reference number respectively is shown next to each entry. B) Putative CtrA-binding site of Jorvik shows similarities to those identified in *Rhodobacte*r species.

Table S1. Proteins encoded by phage Jorvik and close homologs identified using blastp, matrix BLOSUM=45, related to Figure 1. Genes in bold are not encoded in Jorvik and are annotated according to homologous phages. Proteins without the accession number were identified in this study and their sequence and coordinates can be found in **Data S2**.

Rhodobacter phage Jorvik [OP588643]					*Marinomonas* phage YY [BK062760]			*Marinosulfonomonas* sp. PRT-SC04 [JPUR01000104]			*Neotabrizicola shimadae* strain N10 [CP069370.1]			*Pseudophaeobacter* sp. MA21411-1 [JAJQRG010000003.1]			*Rhodobacteraceae* bacterium [WTAL01000162]			*Tropicibacter* sp. LMIT003 [RCTZ02000001]		
Operon	protein	putative function	length [AA]	Pfam / PM2 hit	protein	query [%]	identity [%]	protein [accession]	query [%]	identity [%]	protein [accession]	query [%]	identity [%]	protein [accession]	query [%]	identity [%]	protein [accession]	query [%]	identity [%]	protein [accession]	query [%]	identity [%]
	gp1	HTH domain-containing protein	74	PF08279	gp20	-	-	KPU84703	55	40	Nss3	98	36	MCD9147859	82	38	NOR63035	59	30	WP_121630103	-	-
Replication (O_{fwd1})	gp2	replicase	212	PF13730	gp19	-	-	KPU84702	50	32	Nss2	45	30	Pss1	64	32	NOR63034	-	-	WP_121630102	67	34
	-	**DNA-binding protein**	**121**	-	-	-	-	**KPU84701**	-	-	-	-	-	-	-	-	-	-	-	-	-	-
	-	**unknown**	**103**	-	-	-	-	**KPU84700**	-	-	-	-	-	-	-	-	-	-	-	-	-	-
	-	**unknown**	**219**	-	**gp18**	-	-	Mas6	-	-	QYZ71231	-	-	MCD9147860	-	-	-	-	-	-	-	-
	-	**DNA helicase**	**78**	-	**gp17**	-	-	-	-	-	-	-	-	-	-	-	-	-	-	-	-	-
	gp3	peptidase M15 family	184	PF08291	gp16	75	43	Mas5	87	51	QYZ71230	58	60	MCD9147861	84	55	NOR63033	-	-	WP_121630101	-	-
Packaging (O_{rev1})	gp4	unknown	30	-	-	-	-	-	-	-	-	-	-	-	-	-	NOR63032	-	-	-	-	-
	gp5	FtsK ATPase P9	199	PF13401	gp15	92	38	KPU84699	89	34	QYZ71229	92	40	MCD9147862	93	34	NOR63031	91	34	WP_121630100	91	36
	gp6	virion membrane assembly	156	-	gp14	82	26	KPU84698	85	28	QYZ71228	76	30	MCD9147863	54	26	NOR63030	91	29	WP_121630099	81	24
	gp7	P15-like repressor	149	PF15943	gp13	62	33	KPU84697	62	24	QYZ71227	48	35	Pss2	63	26	NOR63029	44	35	WP_121630098	62	35
	gp8	membrane protein P7	34	-	gp12	91	45	Mas4	97	36	Nss1	61	43	Pss3	-	-	Rhb2	94	44	Trs1	94	41
	gp9	major capsid protein P2	328	PF18628	gp11	77	28	KPU84696	90	24	QYZ71226	98	38	MCD9147864	82	35	NOR63028	93	33	WP_121630097	84	33
	gp10	membrane protein P3	102	-	gp10	98	38	KPU84695	94	40	QYZ71225	61	49	MCD9147865	70	29	NOR63027	97	31	WP_121630096	100	34
	-	**membrane protein P4**	**68**	**Q37958**	-	-	-	**KPU84694**	-	-	-	-	-	-	-	-	-	-	-	-	-	-
	gp11	membrane protein P8	75	Q9XJR5	gp9	92	31	-	-	-	QYZ71224	40	57	MCD9147866	92	34	NOR63026	89	40	WP_121630095	89	24
	gp12	N-acetyltransferase	134	PF13673	gp8	93	34	KPU84693	94	29	QYZ71223	87	45	MCD9147867	74	46	NOR63025	88	40	WP_121630094	60	46
Structural (O_{fwd2})	gp13	virion protein P10	179	Q9XJR4	gp7	87	27	KPU84692	95	27	QYZ71222	87	32	MCD9147868	98	28	NOR63024	96	28	WP_121630093	95	28
	gp14	spike protein P1	248	-	gp6	72	18	KPU84691	93	22	QYZ71221	83	28	MCD9147869	23	31	NOR63023	76	23	WP_121630092	87	25
	gp15	virion-associated lysin Slt	231	PF19489	gp5	54	34	Mas3	90	28	QYZ71220	60	46	MCD9147870	47	40	NOR63022	97	34	WP_121630091	97	30
	gp16	unknown	64	-	gp4	-	-	Mas2	-	-	QYZ71219	-	-	MCD9147871	-	-	NOR63021	-	-	WP_121630090	-	-
	gp17	unknown	63	-	gp3	-	-	KPU84690	-	-	QYZ71218	-	-	MCD9147872	-	-	NOR63020	-	-	-	-	-
	gp18	unknown	119	-	gp2	-	-	Mas1	-	-	QYZ71217	-	-	Pss4	-	-	Rhb1	-	-	-	-	-
	gp19	unknown	64	-	-	-	-	-	-	-	QYZ71216	-	-	-	-	-	-	-	-	-	-	-
	gp20	holin	77	PF11351*	gp1	70	39	KPU84689	71	36	QYZ71215	77	44	MCD9147873	72	32	NOR63019	59	36	WP_121630089	84	38

**annotated using the sequence of Marinomonas phage YY homolog; AA, amino acid; gp, gene product; Pfam / PM2 hit, HHpred-identified hits in Pfam database or among UniProtKB proteins of phage PM2*

Table S2: Comparison of phage Jorvik and PM2 gene products using HHpred pairwise alignment [S7], related to Figures 1 and 6. Hits with E-value lower than 0.01 and Score higher than 20 were considered significant (highlighted in grey).

Jorvik gene product	Aligned region (protein length)	PM2 gene product	Aligned region (protein length)	Function	AA identity [%]	E-value	Score
gp5	8-193 (199)	P9	11-202 (218)	FtsK ATPase P9	20	6.70E-20	96.54
gp6*	71-85 (156)	gp-h	63-77 (143)	virion membrane assembly	20	2.50E-01	12.48
gp7	13-38 (149)	P15	18-44 (157)	P15-like repressor	19	1.10E-04	28.23
gp8	1-19 (34)	P7	1-19 (34)	membrane protein P7	32	1.50E-02	12.27
gp9	37-327 (328)	P2	1-266 (269)	major capsid protein P2	19	9.30E-55	371.76
gp10	5-39 (102)	P3	2-36 (104)	membrane protein P3	23	2.40E-02	16.6
gp11	33-73 (75)	P8	42-74 (75)	membrane protein P8	24	5.40E-07	35.52
gp13	113-148 (179)	P10	100-135 (279)	virion protein P10	8	4.70E-05	37.04
gp14*	1-74 (248)	P1	1-79 (335)	spike protein P1	7	5.70E-02	20
gp15	1-15 (231)	P5	1-16 (160)	virion-associated lysin Slt	13	2.10E-02	16.34
gp20	42-59 (77)	gp-k	1-18 (53)	holin	11	4.80E-02	11.48

**function further confirmed by AlphaFold2 structure prediction (see main text for details).*

Table S3. Host range of phage Jorvik variants on different *R. capsulatus* SB1003 mutants, related to Table 1.

SB1003 mutant	Reference	Jorvik1			Jorvik2		
Δ*rcc002622*	This study	+	+	++	++	++	++
Δ*rcc002623*	This study	++	++	++	++	++	++
Δ*rcc000280*	[S8]	++	++	++	++	++	++
Δ*ctrA*	[S5]	-	-	-	-	-	+
Δ*dprA*	This study	++	-	+	++	+	++
Δ*g16*	[S9]	++	+	+	++	++	++
WT	[S10]	++	++	++	++	++	++
Δ*ctrA*_pCM66T_Ppuf_*ctrA*	[S5]	+	+	-	+	+	++
Δ*ctrA*_pCM66T_Ppuf_*ctrA*D51A	[S5]	-	-	-	-	+	+
Δ*ctrA*_pCM66T_Ppuf_*ctrA*D51E	[S5]	-	-	+	+	+	+
WT_pCM66T_Ppuf_*ctrA*	[S5]	++	++	++	++	++	++
WT_pCM66T_Ppuf_*ctrA*D51A	[S5]	++	++	++	++	++	++
WT_pCM66T_Ppuf_*ctrA*D51E	[S5]	+	+	++	++	++	++

+, impaired plaques; ++ normal plaques; -, no plaques; Δ, deletion; Ppuf, promoter of puf operon; WT, wild type

Table S4. Taxonomy ranking of sequences containing major capsid protein P2 and packaging ATPase P9 homologs of phage Jorvik within 10kb, related to Figure 5. Selected representatives of each taxon were analysed in detail to confirm the presence of other phage-like proteins.

	Taxonomy rank	N(hits)	Max length of the contig	Representative	Nucleotide ID	Putative phage start	Putative phage end	N(ORF)	Phage PM2 hits*	Replicase type*
Proteobacteria	Alphaproteobacteria	44	>100 000	*Pseudophaeobacter* sp. MA21411-1	JAJQRG010000003	439166	448708	21†	P1, P2, P3, P8, P9, P10, P15	J
	Betaproteobacteria	152	>100 000	*Burkholderia cepacia*	CP013375.1	2000802	2016439	22	P1, P2, P8, P9, P10, P12, P14, P15	C
	Gammaproteobacteria	622	>100 000	*Vibrio cholerae*	CP024083.1	436074	449159	28	P1, P2, P3, P5, P6, P8, P9, P10, P14, P15, P16, gp-h	C
	Deltaproteobacteria	1	7679	*Desulfuromonadales* bacterium C00003093	MAXS01000146.1	1	7679	12	P1, P2, P3, P5, P9, P10, gp-h	A/T
	Epsilonproteobacteria	1	8665	*Sulfurimonas* sp. UBA10385	DLUC01000089.1	1	8665	16	P1, P2, P3, P8, P9, P10, gp-k	J
	Unclassified proteobacteria	1	22929	Proteobacteria bacterium CAG:495	CAZU010000021.1	1	9651	17	P1, P2, P9, P10, P14, P16	J
DJR viruses	*Tectiliviricetes*	12	12527	*Pseudoalteromonas* phage PM2	AF155037.1	1	10079	22	NA	C
	unclassified *Varidnaviria*	1	10075	*Marinomonas* phage MfV	MW618650.1	1	10075	20	P1, P2, P8, P9, P10, P12, P14, gp-k	C
PVC bacteria	Verrucomicrobia	8	>100 000	*Opitutaceae* bacterium TAV5	CP007053.1	3882770	3903424	31	P2, P3, P9, P10, P12	C
	other PVC group bacteria	2	10767	*Planctomycetes* bacterium RBG_13_46_10	MHYD01000052.1	1	10767	21	P1, P2, P3, P8, P9, P14	NA
Other bacteria	Firmicutes	3	15263	*Exiguobacterium antarcticum* DSM 14480	NZ_JMKS01000002.1	1	14829	26	P2, P8, P9, P14, P15	A/T
	Others	6	43089	*Nitrospirae* bacterium GWB2_47_37	MHDU01000077.1	1	10596	23	P1, P2, P9, P10, P15, P16	J

**estimated by HHpred; †re-estimated by GeneMark; DJR, double jelly roll viruses; AT, Autolykiviridae/Tectiviridae-like replicase; J, phage Jorvik-like replicase; C, Corticoviridae-like replicase; NA, not applicable; PVC, Planctomycetota, Verrucomicrobiota, and Chlamydiota superphylum*

Table S5: Oligonucleotides used in this study, related to STAR Methods.

Purpose	Name	Sequence	Function
Generating knock outs in *R. capsulatus*	DprA ext F	CGACTCTAGAGGATCTCGAGGACGGCGTGCTGG	*dprA*-flanking DNA #1
	DprA inv R	GGGAATCAGGGGATCCGGAGAAACGAGAGCTCG	
	DprA inv F2	AACAATTCGTTCAAGCCTGTCACGCGAAGTGATC	*dprA*-flanking DNA #2
	DprA ext R2	CGGTACCCGGGGATCGACTTCCCATTTCATCTCG	
	2622_3 ext F	CGACTCTAGAGGATCGCGAAAATGATCACC	2622_3 ext F was used to make flanking DNA #1 for both *2622* and *2623* together with the relevant R primers
	2622 inv R	GGGAATCAGGGGATCGACATTGTCTCTCCGAATGG	
	2623 inv R	GGGAATCAGGGGATCATCGACACGCCGAACTGAG	
	2622 inv F	AACAATTCGTTCAAGCGGATCTGACCTGAGGCC	2622_3 ext R was used to make flanking DNA #2 for both *2622* and *2623* together with the relevant F primers
	2623 inv F	AACAATTCGTTCAAGCCATCGGTGATCGCGATG	
	2622_3 ext R	CGGTACCCGGGGATCGATGGAGGATCTGTTGGCAC	
	Gent F	GATCCCCTGATTCCCTTTGT	Gentamicin cassette
	Gent R	CTTGAACGAATTGTTAGG	
Expression of putative lytic genes of Jorvik	M15 F	TCCAGGGACCAGCAATGAGCCGCGACCCGATCC	Amplification of gene *M15*
	M15 R	TGAGGAGAAGGCGCGTCAAGCCCGCTTCCGTTTCAGC	
	Slt F	TCCAGGGACCAGCAATGCGCGGGATTGTCATTCTG	Amplification of gene *slt*
	Slt R	TGAGGAGAAGGCGCGTCACGCCATCGCCCCTTG	
	Lic F	CGCGCCTTCTCCTCACATATGGCTAGC	Linearisation of pETYSBLIC3c
	Lic R	TTGCTGGTCCCTGGAACAGAACTTCC	

Supplementary References

S1. Jumper, J., Evans, R., Pritzel, A., Green, T., Figurnov, M., Ronneberger, O., Tunyasuvunakool, K., Bates, R., Žídek, A., Potapenko, A., et al. (2021). Highly accurate protein structure prediction with AlphaFold. Nature *596*, 583–589. 10.1038/s41586-021-03819-2.

S2. Holm, L. (2020). DALI and the persistence of protein shape. Protein Sci *29*, 128–140. 10.1002/pro.3749.

S3. Carnoy, C., and Roten, C.-A. (2009). The dif/Xer Recombination Systems in Proteobacteria. PLoS One *4*, e6531. 10.1371/journal.pone.0006531.

S4. Huber, K.E., and Waldor, M.K. (2002). Filamentous phage integration requires the host recombinases XerC and XerD. Nature *417*, 656–659. 10.1038/nature00782.

S5. Fogg, P.C.M. (2019). Identification and characterization of a direct activator of a gene transfer agent. Nature Communications *10*, 595. 10.1038/s41467-019-08526-1.

S6. Hernández-Valle, J., Sanchez-Flores, A., Poggio, S., Dreyfus, G., and Camarena, L. (2020). The CtrA Regulon of Rhodobacter sphaeroides Favors Adaptation to a Particular Lifestyle. J Bacteriol *202*, e00678-19. 10.1128/JB.00678-19.

S7. Zimmermann, L., Stephens, A., Nam, S.-Z., Rau, D., Kübler, J., Lozajic, M., Gabler, F., Söding, J., Lupas, A.N., and Alva, V. (2018). A Completely Reimplemented MPI Bioinformatics Toolkit with a New HHpred Server at its Core. J Mol Biol *430*, 2237–2243. 10.1016/j.jmb.2017.12.007.

S8. Ding, H., Grüll, M.P., Mulligan, M.E., Lang, A.S., and Beatty, J.T. (2019). Induction of Rhodobacter capsulatus Gene Transfer Agent Gene Expression Is a Bistable Stochastic Process Repressed by an Extracellular Calcium-Binding RTX Protein Homologue. J Bacteriol *201*, e00430-19. 10.1128/JB.00430-19.

S9. Sherlock, D., and Fogg, P.C.M. (2022). Loss of the Rhodobacter capsulatus Serine Acetyl Transferase Gene, cysE1, Impairs Gene Transfer by Gene Transfer Agents and Biofilm Phenotypes. Applied and Environmental Microbiology *88*, e00944-22. 10.1128/aem.00944-22.

S10. Strnad, H., Lapidus, A., Paces, J., Ulbrich, P., Vlcek, C., Paces, V., and Haselkorn, R. (2010). Complete genome sequence of the photosynthetic purple nonsulfur bacterium Rhodobacter capsulatus SB 1003. J Bacteriol *192*, 3545–3546. 10.1128/JB.00366-10.
